# The Biochemistry and Evolution of the Dinoflagellate Nucleus

**DOI:** 10.3390/microorganisms7080245

**Published:** 2019-08-08

**Authors:** Sebastian G. Gornik, Ian Hu, Imen Lassadi, Ross F. Waller

**Affiliations:** 1 Centre for Organismal Studies (COS), Universität Heidelberg, 69120 Heidelberg, Germany; 2 Department of Biochemistry, University of Cambridge, Cambridge CB2 1QW, UK

**Keywords:** dinoflagellate phylogeny and evolution, dinokaryon, DVNP, HLP, histone, cholesteric liquid crystalline DNA

## Abstract

Dinoflagellates are known to possess a highly aberrant nucleus—the so-called dinokaryon—that exhibits a multitude of exceptional biological features. These include: (1) Permanently condensed chromosomes; (2) DNA in a cholesteric liquid crystalline state, (3) extremely large DNA content (up to 200 pg); and, perhaps most strikingly, (4) a deficit of histones—the canonical building blocks of all eukaryotic chromatin. Dinoflagellates belong to the Alveolata clade (dinoflagellates, apicomplexans, and ciliates) and, therefore, the biological oddities observed in dinoflagellate nuclei are derived character states. Understanding the sequence of changes that led to the dinokaryon has been difficult in the past with poor resolution of dinoflagellate phylogeny. Moreover, lack of knowledge of their molecular composition has constrained our understanding of the molecular properties of these derived nuclei. However, recent advances in the resolution of the phylogeny of dinoflagellates, particularly of the early branching taxa; the realization that divergent histone genes are present; and the discovery of dinoflagellate-specific nuclear proteins that were acquired early in dinoflagellate evolution have all thrown new light nature and evolution of the dinokaryon.

## 1. Introduction

Dinoflagellates are amongst the most abundant marine organisms with an estimated 8000 species of which approximately 2400 are named to date [1,2]. As photosynthetic organisms they contribute a substantial fraction of global carbon fixation, and as essential symbionts of corals they maintain coastlines and coral reef ecosystems throughout the tropics. Some form toxic algal blooms hundreds of kilometers long, and others have evolved as fish and shellfish parasites with significant impact to food security and economies.

The dinoflagellate phylum together with the ciliate and apicomplexan phyla forms the infrakingdom Alveolata. A unifying feature of alveolates is a common cell structure—a continuous layer of flattened vesicles underneath their plasma membrane called alveoli. Sister taxa dinoflagellates and apicomplexans share several further derived features. Both have unusual mitochondria that have highly reduced genomes [3] and lack canonical enzyme complexes such as those for pyruvate dehydrogenase and complex 1 of the electron transport chain [4]. While most apicomplexans are parasitic and many dinoflagellates are photosynthetic, the basal members of both phyla display a number of similarities such as heterotrophy and a propensity to interact with other organisms both as symbionts and parasites [5,6].

Despite shared characteristics with apicomplexans, dinoflagellates have taken a conspicuous evolutionary trajectory with respect to their nuclear biology, departing from many of the norms of the typical eukaryotic nuclei that are otherwise found in apicomplexans. The dinoflagellate nucleus is so unusual that the term ‘dinokaryon’ was coined which, for a time, was thought to represent a missing link as an intermediate ‘mesokaryotic’ state between prokaryotic DNA coils and true eukaryotic nuclei [7]. Molecular phylogenetics, however, clearly shows that the dinoflagellates are true eukaryotes and, therefore, that their aberrant nuclei are a derived from the canonical form [2,8,9].

In this review, we consider the advances in knowledge of the biochemistry and the evolution of dinoflagellate nuclei, with an emphasis on recent scientific progress.

## 2. Improved Dinoflagellate Phylogenies Provide a Platform for Better Understanding Dinokaryon Development

Until the application of molecular phylogeny in the 1990s, the systematics of dinoflagellates was derived from fossil records and the tabulation of morphological and physiological traits of extant species. This resulted in considerable uncertainty both of the relationships within dinoflagellates as well as those to major sister lineages. This changed with the introduction of molecular phylogenies. Dinoflagellate relationships are now well resolved, and we know with certainty that dinoflagellates, together with the ciliates and apicomplexans, belong to the Alveolata forming one of the most robustly supported eukaryotic supergroups [8,10,11,12,13,14,15]. Despite these advances, until recently, the evolutionary history of some of the major dinoflagellate lineages was still poorly resolved [14]. This changed with the use of phylotranscriptomics drawing on extensive RNA-seq data from diverse dinoflagellates. Initially, the deeper branches of the dinoflagellate lineage came into focus with use of ribosomal proteins [8]. Subsequent expanded transcriptome datasets using well-defined conserved eukaryote proteins generated a strongly supported dinoflagellate phylogeny representing major taxa [2].

**Figure 1:**
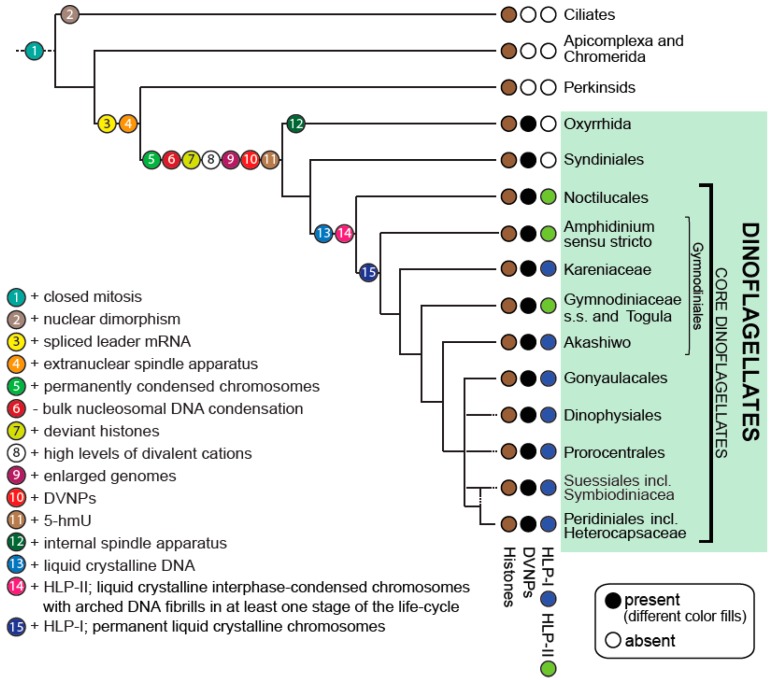
Nuclear character state evolution in dinoflagellates. Model depicting a consensus phylogeny of dinoflagellates and the progression of nuclear character state changes. Dotted branches indicate uncertain placements of taxa. Modified from Janouškovec et al. (2017) [2] and extended.

The current resolution of dinoflagellate relationships allows inference of ancestral traits and the evolution of morphological and biochemical features with a certainty that was not previously possible. These have included traits such as bioluminescence and the evolution of the cellulosic cell wall of the thecate dinoflagellates [2]. Importantly, it provides a platform to consider the progression of changes that have led to the most unusual dinoflagellate trait, the dinokaryon (see Figure 1).

## 3. Dinoflagellate Nuclear Biochemistry Is Highly Unusual

The broad evolutionary history of eukaryotes suggests that the last common ancestor of the alveolates possessed a typical nucleus [16,17,18]. The nuclear morphology, organization, and biochemistry of extant alveolates, however, exhibits an unusually large number of aberrant features and a high degree of plasticity. Ciliates evolved to contain two dimorphic nuclear forms: One diploid, transcriptionally silent germ line micronucleus (the so-called the ‘generative nucleus’); and at least one somatic, often polyploid macronucleus (the so-called ‘vegetative nucleus’) per cell. The transcriptionally active macronuclear DNA is derived from the micronucleus but is vastly rearranged after mitosis [19]. Apicomplexans have retained a more ‘classic’ nuclear arrangement; however, they have drastically reduced the size of their genomes in response to parasitism when compared to other, non-parasitic eukaryotes [19,20].

All three phyla exhibit a closed mitosis where the nuclear envelope remains intact during DNA replication and chromosome segregation [5,21,22,23]. Ciliates and apicomplexans possess an internal spindle apparatus. The mitotic spindle of most dinoflagellates, however, is extra-nuclear [5,22,24]. Centrioles are seemingly absent in ciliates and dinoflagellates, but they are present in apicomplexans [25,26]. Exceptions to these are the dinoflagellate *Oxyrrhis marina*, which has an internal spindle [27] and the syndinean dinoflagellates, which possess centrioles [28]. Clearly, the nuclei of the alveolates are extremely diverse and highly unusual when compared with canonical nuclei of other more studied model systems. 

In addition to the diversity of nuclear organization and division in Alveolates, the biochemistry of dinoflagellate nuclei is particularly unusual. The dinoflagellate nucleus contains: (i) An extremely large DNA content (up to 200 pg per nucleus); (ii) nuclear DNA that contains large amounts of Mg^2+^ and Ca^2+^ cations and various transition metal ions; (iii) abundant nucleotide modifications such as 5-hydroxymethyluracil, which replaces up to 70% of thymidine; (vi) a striking deficit of histones that is accompanied with a skewed nuclear DNA-to-protein ratio of up to 10:1 (rather than the typical eukaryotic 1:1 ratio); and (v) novel nuclear proteins, namely: Dinoflagellate ‘histone-like proteins’ (type-I and type-II HLPs) perhaps derived from bacteria and a family of DNA-binding proteins of putative viral origin called ‘dinoflagellate/viral nucleoproteins’ (DVNPs) ([2] and references therein). The interplay of all these characters presumably result in the characteristic features of the dinoflagellate nucleus: Permanently condensed chromosomes of liquid crystalline DNA.

### 3.1. Extremely Large Nuclear DNA Content

Dinoflagellates can possess enormous amounts of DNA per nucleus, only surpassed by the fathomless genomes of some amoeba [29]. Dinoflagellate DNA content per cell measured using fluorescent dyes can range from 1.5 to 4 pg in the genus *Symbiodinium* to more than 100–200 pg in species such as *Alexandrium tamarense* and *Prorocentrum micans* ([30] and references therein). However, smaller or larger dinoflagellate genomes might await discovery still [31]. The human nucleus, for comparison, contains approximately 3 pg of DNA. A positive correlation exists between dinoflagellate genome size and cell size, as larger species tend to possess larger genomes [30]. However, only a small fraction of all extant dinoflagellate species has been analyzed for their DNA content to date and it remains to be seen whether the correlation can be verified when more species are sampled. Interestingly, not only do most dinoflagellates have enormous genomes, but they also often possess a large number of chromosomes per nucleus with, for example, *Alexandrium fundyense* maintaining more than 100 chromosomes per cell nucleus [5,22]. Other more basal species such as *Hematodinium*, however, harbor only four chromosomes [32]. Although unusual for single-celled protists, such large chromosome numbers are not unprecedented within the eukaryotes. The highest number of chromosomes for a eukaryote to date is 1440 in the Adder’s-tongue fern *Ophioglossum reticulatum* [33]. The silver crucian freshwater carp *Cyprinus auratus gibelio* possesses up to 162 chromosomes [34] and the red viscacha rat *Tympanoctomys barrerae* possesses 102 chromosomes [35]. The closely related apicomplexan parasites *Plasmodium falciparum* and *Babesi bovis*, on the other hand, contain only 14 chromosomes [36] and four chromosomes [37,38], respectively. Perkinsids, which are basal to dinoflagellates but still possess canonical nuclei, also have few chromosomes; for example, nine chromosomes in *Perkinsus olseni* ranging in size from 0.15 Mb to 6.5 Mb [39,40].

### 3.2. High Concentrations of Bivalent Cations and Transition Metals

Using analytical electron microscopy, Herzog and Soyer (1983) showed that dinoflagellate chromosomes contain the bivalent cations of calcium (Ca^2+^) and magnesium (Mg^2+^) [41]. Herzog and Soyer also demonstrated that these ions are necessary for the stabilization of dinoflagellate chromosomes—a feature that was later revealed to be important for the chromosome condensation of all eukaryotes [42,43]. Further, in a more recent study employing secondary ion mass spectrometry, it has been shown that in the dinoflagellate *P. micans* Mg^2+^ and Ca^2+^ cations occur at a concentration of approximately 0.6 cations per base pair and a ratio of 3:1 within the nucleus [44]. By contrast, in human cells one Ca^2+^ cation occurs every 12.5–20 bases and one Mg^2+^ cation only every 20–30 bases [42]. Interestingly, the per-base cation values measured in *P. micans* are close to values obtained in vitro for complete neutralization of the intrinsic DNA charge and maximal condensation of naked DNA (3:1 ratio of Mg^2+^ to Ca^2+^ at 0.63 cations per base pair) [45]. Based on these data, Koltover et al. (2000) suggested that in *P. micans* DNA is condensed as a naked molecule (in the absence of histones) purely by the direct action of the abundant bivalent cations Mg^2+^ and Ca^2+^. The authors further speculate that this mode of DNA condensation is typical for all dinoflagellates. While subsequent reports of some presence of histones and other nuclear proteins in dinoflagellates (which will be discussed further below) suggest that this model is overly simplistic, it nevertheless demonstrates a potentially important role of bivalent cations in the nuclei of dinoflagellates when compared to other eukaryotic groups. Kearns and Sigee (1980) also demonstrated the presence of the transition metals copper (Cu), zinc (Zn), iron (Fe), and nickel (Ni) in chromosomes of a number of dinoflagellate taxa namely *P. micans*, *Amphidinium carterae*, *Peridinium faroense*, *Ceratium hirundinella*, and *Glenodinium foliaceum* comprising members of most dinoflagellate orders [46]. It is thus evident that bivalent cations and transition metals play an important role in the nuclei of all core dinoflagellates. However, the amounts of divalent cations and transition metals in basal dinoflagellate taxa such as *Oxyrrhis* or *Hematodinium* sp. are not known to date, and further research is needed to elucidate the evolution of metal content in dinoflagellate nuclei.

### 3.3. High Amounts of DNA Modifications

Genomic DNA in all organisms is known to contain numerous modified bases, which have various functions although many of which are still unknown [47]. The best-examined modified bases are 5-methylcytosine (5-mC) and 5-hydroxymethylcytosine (5-hmC), which have been identified as important epigenetic modifications of genomic DNA in many eukaryotes [47,48,49]. These modifications are enzymatically derived; however, others are caused by physical DNA damage caused by both endogenous and exogenous sources [50]. In mammals, for example, 5-hydroxymethyluracil (5-hmU) is conferred by ionizing radiation, either through direct energy adsorption or indirectly through the generation of free radicals that elicit damage via oxidative stress [51]. In a small number of bacteriophages, all thymine bases are substituted by 5-hmU during replication potentially allowing avoidance of DNA degradation by the host [50,52]. Generation of 5-hmU is likely conveyed by members of the ten–eleven translocation (TET) family of enzymes [53]. Not much is known about the function of 5-hmU in nature, but it has been reported to affect protein-binding to DNA in one report [54].

Interestingly, 5-hmU is also a common and apparently conspicuous feature of dinoflagellate nuclear DNA. In dinoflagellates, 5-hmU replaces between 12% and 70% of thymine depending on species [55,56,57]. While the initial data for 5-hmU were based on the buoyant density measures of DNA, the occurrence of 5-hmU was later confirmed by HPLC [58]. Steele and Rae (1980) also showed that 5-hmU is non-randomly distributed in the genome of dinoflagellates and that 5-hmU preferentially replaces thymine in the dinucleotides TA and TC [59]. Unfortunately, no data is available for 5-hmU content in the basal dinoflagellates, so it is unclear when changes to nucleotide modifications occurred in radiation of dinoflagellates. The biological reason for the occurrence of 5-hmU in dinoflagellate nuclear DNA is also unknown, and curiously no further attempts have been made to understand its role to date. As more knowledge of the nuclear organization and nuclear biochemistry of dinoflagellates emerges, and with the arrival of new high-throughput sequencing technology that allows genome-wide mapping of 5-hmU-modified loci, this might change in the near future and we might see a rekindling of interest in dinoflagellate 5-hmU.

As well as 5-hmU the nuclear DNA of dinoflagellates, 5-hmC at levels comparable to other eukaryotes is also found [60]. Mendoza et al. (2018) show that two members of the Symbiodiniaceae (*Fugacium kawagutii* and *Breviolum (Symbiodinium) minutum*) evolved cytosine methylation patterns that are unlike any other patterns found in eukaryotes to date [61]. For example, they determine that the relative methylation levels at single CG sites are unimodal rather than bimodal, with most (99.9%) CG sites being highly methylated. Interestingly, they also show that individual cells do not undergo differential methylation in response to environmental stress such as heat. The authors also determine a high degree of cell-to-cell heterogeneity and conclude that in Symbiodiniaceae CGs are by default hypermethylated and not differentially methylated. Thus, a definitive role for the observed unusual methylation patterns at CG sites in these genomes remains elusive.

### 3.4. Non-Canonical, Deviant Histones

The nearest relatives of dinoflagellates, namely apicomplexans and ciliates, pack their DNA into regular repeating units called nucleosomes similar to most other eukaryotes. These “beads on a string” are then organized into higher-order chromatin structures [62,63,64]. Nucleosomes are formed by the interplay of histones and nuclear DNA in such ways that 150–200 base pairs of DNA are wrapped approximately 1.7 times around an octamer composed of two copies of the canonical histones H2A, H2B, H3, and H4 in an H3–H4 and H2A–H2B dimeric arrangement. During DNA replication, histones are first displaced and then incorporated into both the parental and the newly synthesized strand in a tightly controlled process called replication-coupled nucleosome assembly [65]. 

Within nucleosomes, N- and C-terminal modifications in histone tail regions act synergistically or antagonistically to modulate interactions with chromatin-associated proteins and thus dictate dynamic transitions between accessible, open chromatin (transcriptionally active) and compacted chromatin (or transcriptionally silent) states. These conventional eukaryotic processes, that extend the information potential of eukaryotic genomes, have been coined the ‘histone code’ [66,67]. Universal non-canonical histone variants exist alongside the canonical histones in all eukaryotes, including ciliates and apicomplexans [64,68], and are incorporated, exchanged, or displaced throughout the cell cycle in a replication-independent manner and, in metazoans, are also found associated with certain developmental stages and disease [65]. The histone variants are derived from the canonical forms, some even before the divergence of extant eukaryotes, and fulfill important functions in DNA repair, chromosome segregation, transcription initiation, and termination [62,68]. The centromere-specific H3 variant (CenH3s), for example, is essential for the assembly of the kinetochore [62,69]. Another histone variant, H2A.Z, is often found on either side of transcription start sites, where it promotes efficient RNA polymerase II recruitment [70]. In the apicomplexan *P. falciparum*, H2A.Z is enriched in the promoter region of active var genes important for host immune evasion [71]. The histone variant H2A.X is implicated in DNA repair [72], while H3.3 is associated with both replication-dependent and replication-independent nucleosome assembly as well as epigenetic memory [73]. However, many more variants exist and much about their functions remains elusive and needs clarification [62]. The functional of the canonical histones in bulk DNA management means that their expression is tied to the cell cycle in an unusual way. In some protozoa and all metazoans, although not in plants and fungi, these histone mRNAs are not polyadenylated and instead mature histone mRNA contain a unique stem–loop structure close to the 3’ end, which is generated by cleavage using a machinery involving the U7 snRNP and protein factors such as the stem–loop binding protein (SLBP). Degradation of these unusual histone mRNAs requires initial translation and presence of the stem loop. The molecular mechanisms that underlie histone mRNA degradation, however, are not yet understood but it is assumed that tight regulation of SLBP levels effectively restrict histone mRNA accumulation to S-phase [74]. By contrast, the synthesis of the variant, replication-independent histones, are encoded by polyadenylated mRNAs (for a detailed review of the birth and death of histone mRNAs see [74,75]).

Remarkably, the dinoflagellates seemingly have abandoned canonical bulk, histone-mediated nucleosomal compaction of chromatin entirely. Until very recently, dinoflagellates were thought to lack histones altogether, an observation, which was supported by a large number of biochemical, histological, and microscopic observations across a large diversity of different dinoflagellate taxa. For example, (1) the chromatin spreading technique, which otherwise visualizes nucleosomal particles in eukaryotes, showed only smooth strands of naked DNA in *P. micans* [76,77], *Crypthecodinium cohnii* [78,79], and many other dinoflagellate taxa (see [80,81] and references therein); (2) acid or salt extracts, which in other eukaryotes recover all four core histones, lack typical histone banding patterns on SDS-PAGE gels (e.g., [82,83,84,85,86]); (3) the ratio of basic protein to DNA, which is roughly 1:1 in other eukaryotes and indicative for the abundance of histones in their nuclei, is skewed closer to 1:10 or less and most dinoflagellate nuclei do not stain with the basic-protein-sensitive alkaline fast green stain [5,81,87]; (4) in contrast to the chromatin of other eukaryotes, the chromatin of dinoflagellates does not confer nucleosome-mediated protection against nuclease digestion [32].

Despite the initial biochemical evidence for no histones in dinoflagellates, subsequent molecular data revealed that dinoflagellates do possess the histones H2A, H2B, H3, and H4, however, of unusually low abundance and divergent sequence. A ‘rediscovery’ of dinoflagellate histones came through expressed sequence tag (EST) transcriptome data for *Pyrocystis lunula* and *A. tamarense* [88,89]. Afterward, with (i) the arrival of next-generation sequencing technologies allowing much deeper sequencing of dinoflagellate transcriptomes, (ii) the identification of the dinoflagellate spliced leader, which allows the unambiguous identification of dinoflagellate transcripts, and (iii) an increased number of dinoflagellate RNA-seq projects, all four histone types were identified bioinformatically in a large number of dinoflagellates [32,90,91]. These studies also reveal that expression levels are generally low. Expression levels of histone transcripts have been estimated in *Lingulodinium polyedrum* using RT-qPCR. Results indicate that histones are expressed at roughly 25 times lower levels than in other eukaryotes [90]. Using biochemical methods, it was also shown that histone proteins are low in abundance and that bulk nucleosomes are absent [32,90]. Interestingly, there is no evidence for an RNA stem–loop structure close to the 3′ end of dinoflagellate histone mRNAs and transcripts are generally polyadenylated, like the bulk of dinoflagellate mRNA and also carry spliced leader sequences [92,93]. This suggests that in dinoflagellates histone expression is independent of the cell cycle. By contrast, the close relative of dinoflagellates, *Perkinsus marinus*, shows canonical levels of histones and also contains 3′ RNA stem–loop structures, tying histone expression to the cell cycle [32,94].

Dinoflagellate histone sequences are readily identified and categorized into four known histone families H2A, H2B, H3, and H4 through their highly conserved histone-fold regions [32]. However, the high divergence of dinoflagellate histones together with an uncertainty of function precludes any further classification, such as variant types, with any certainty. With the exception of the H3 histone, all dinoflagellate histones, possess long, extended N-termini, which are often highly enriched in charged amino acids arginine and lysine [32]. Furthermore, by sequence similarity, dinoflagellate histones are more variable amongst dinoflagellate taxa than other eukaryotic histones are across different phyla and kingdoms. Despite this, Roy et al. (2012) and Bayer et al. (2012) and, to some extent, Marinov and Lynch (2016) attempted to further classify the dinoflagellate histones by comparing them to eukaryotic histones and their variants using phylogenetic methods [90,91,95]. Hence, they have putatively identified H2A.Z and H2A.X in *L. polyedrum* and *Symbiodinium microadriaticum* and *B. (S.) minutum* (both Symbiodiniaceae) as well as H3.3 and a CenH3 in *Symbiodinium* and *Breviolum* only. Without knowledge of histone location and function in the dinokaryon, we feel that this classification is premature. For example, while in most eukaryotes, histone H3.3 differs from canonical H3 by only four amino acids with a maximum difference of 16 changes found in *Tetrahymena thermophila* [62,96], *Symbiodinium* H3.3 (contig kb8_rep_c1944 in Bayer et al. (2012) [91]) differs from *Symbiodinium* H3 (contig kb8_c18106 in Bayer et al. (2012) [91]) by 65.5% (79 AAs over the alignable region) and possesses an additional 67 AAs at the C-terminus. Further, the long branches in the phylogenetic trees of dinoflagellate histones presented in both studies indicate considerably greater protein evolution compared to other eukaryotic histone counterparts.

In conclusion, current data show that core histones are readily identified in all dinoflagellates sequenced to date and that these histones typically occur as multiple variants. Known functional variants such as H2A.Z, H2A.X, and H3.3 may also exist. Nevertheless, since dinoflagellates histones are all highly divergent with a tendency to frequently lose key residues that are otherwise highly conserved, it cannot be excluded that dinoflagellate histones have adopted new, yet unknown roles, especially in the light of their accelerated evolution. Dinoflagellate histones often contain extended N- or C-terminal tails. However, in some species fairly conventional sets of histones co-occur with these length-extended variants. Interestingly, amino acid residues that are involved in transcription elongation control, especially lysine 4 on histone H3 (H3K4) are highly conserved, while histone marks that are associated with chromatin dynamics during mitosis are mostly lost. This suggests that at least parts of the conventional eukaryotic histone code are functional in dinoflagellates and it may be that at least dinoflagellate H3 helps to mediate transcriptional elongation.

### 3.5. Novel Dinoflagellate-Specific Nuclear Proteins

#### 3.5.1. HLPs

A paradigm shift for understanding dinoflagellate nuclear biochemistry came with the discovery of novel nucleoproteins of apparent lateral gain from other organisms. The first described were nuclear proteins of select dinoflagellates that share limited sequence similarity to a family of small, 10 kDA bacterial histone-like proteins (called HU proteins) that are known to coat and thereby compact DNA of many prokaryotes inducing negative supercoiling into circular DNA with the assistance of topoisomerase [97]. Due to their basic nature and presumed function, these proteins were called histone-like basic proteins (HLPs). At least six of these HLPs were observed in the dinoflagellate *C. cohnii* [86] and the coding sequence of four of them were identified (*C. cohnii* histone-like proteins (HCc) HCc1, HCc2, HCc3, and HCc4) [86,97]. HLPs of *C. cohnii* bind to isolated chromatin and are dissociated in 0.6 M NaCl, indicating that their affinity for DNA in vivo is lower than that of core histones [86]. The HCcs locate to the periphery of *C. cohnii* chromosomes, where it has been hypothesized that they might stabilize extra-chromosomal DNA loops from which genes are actively expressed [98,99]. Under atomic force microscopy, HCc3 was shown to induce folded structures with DNA in a concentration-dependent manner [100]. At moderate concentration, DNA bridging and bundling was observed while at high concentrations a stronger DNA condensation was evident. HCc3 also apparently induces DNA condensation in *E. coli* expressing the protein in vivo [101]. In the dinoflagellate *L. polyedrum*, a HLP protein was sequenced and shown to be acetylated in vivo [102]. Furthermore, the protein was found to bind weakly to a specific DNA sequence, which leads the authors to speculate that this protein has a regulatory function at the DNA level. Gradually, HU-like HLPs have been reported in further dinoflagellate taxa [32,103] and Janouškovec et al. (2017) [2] collated presence/absence of HLPs to model their gain and evolution in dinoflagellates. This study revealed that HLPs are absent in all early branching taxa but ubiquitous in core dinoflagellates. They also observed that HLPs in *Noctiluca, Amphidinium, Togula*, and *Gymnodinium* are dissimilar in sequence to HLPs in other core dinoflagellates, and that HLPs form two distinct, mutually exclusive clades (HLP-I and HLP-II) despite their similar length and structure [2]. A definitive role for dinoflagellate HLPs has not been identified yet.

#### 3.5.2. DVNPs

**Figure 2:**
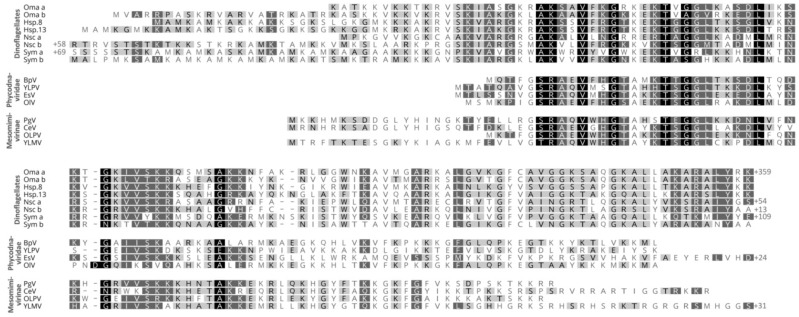
Sequence alignment of dinoflagellate/viral neucleoproteins (DVNPs) from selected species of dinoflagellates, Phycodnaviridae, and the newly proposed Mesomimivirinae. Oma = *Oxyrrhis marina*; Hsp = *Hematodinium* sp.; Nsc = *Noctiluca scintillans*; Sym = *Breviolum (Symbiodinium) minutum*; BpV = *Bathycoccus* sp. virus; YLPV = Yellowstone Lake phycodnavirus; OlV = *Ostreococcus lucimarinus* virus; PgV = *Phaeocystis globosa* virus; CeV = *Chrysochromulina ericina* virus; OLPV = Organic Lake phycodnavirus; YLMV = Yellowstone Lake mimivirus.

A further, novel group of dinoflagellate nuclear proteins was discovered by Gornik et al. (2012) [32]. In the basal parasitic dinoflagellate *Hematodinium* sp., a single protein band was identified from acid-extracted nuclei. This band was shown to correspond to a family of at least 13 highly similar, novel protein-encoding genes. Several studies to date show this new protein occurs in all dinoflagellate taxa, including low-branching taxa that otherwise lack HLPs [2,32,95]. Homologs of this protein do not occur outside of dinoflagellates in the eukaryotic tree of life. However, its presence can be found in two groups of nucleocytoplasmic large DNA viruses, namely the Phycodnaviridae, which predominantly infects photosynthetic marine algae, and Mesomimivirinae, an extended sister group of the mimiviruses previously known as Organic Lake phycodnavirus group (OLPG) and only very recently discovered to host the *DVNP* gene (Figure 2). Both groups have some of the largest and gene-rich viral genomes with hundreds of kilobases of double-stranded DNA. Thus, due to the restricted occurrence of this protein in dinoflagellates and a family of viruses, and its nuclear location in dinoflagellates (see below), the protein family was named dinoflagellate/viral nucleoprotein (DVNP).

**Figure 3:**
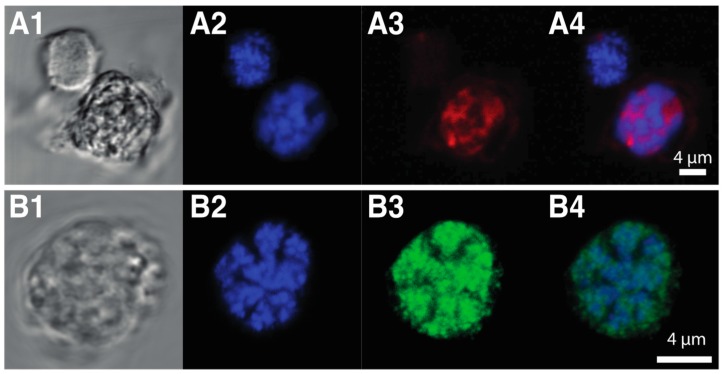
Visualization of two unique characters of dinoflagellate chromatin. (**A**) Assay for Transposase-Accessible Chromatin (ATAC)-see results (**A1**) Brightfield image of two *Hematodinium* sp. cells. (**A2**) DNA staining of the same cells (**A3**) ATAC-see results show that in dinoflagellates at least two chromatin states exist with one nucleus highly stained while a second nucleus is not affected. This resembles accessible euchromatin and inaccessible heterochromatin states found in nuclei of other non-dinoflagellate eukaryotes. (**A4**) Merged image of **A2** and **A3**. (**B**) DVNP immunolocalization. (**B1**) Brightfield image of a *Hematodinium* sp. cell. (**B2**) DNA staining of the same cell. (**B3**) DVNP distribution in the nucleus revealed using a polyclonal anti-DVNP antibody. (**B4**) Merged image of **B2** and **B3**.

Both dinoflagellate and viral DVNPs share a conserved core region, that may play a role in DNA-binding or protein–protein interactions [32]. Dinoflagellate DVNPs also possess a lysine-rich N-terminal extension not found in their viral counterparts (Figure 2 alignment). Furthermore, 2D electrophoresis indicates varying degrees of post-transcriptional modifications of dinoflagellate DVNPs and abundant phosphorylation of DVNPs was confirmed [32]. Immune-fluorescence assays show that DVNPs co-localize with DNA in chromatin in vivo (Figure 3A1–A4). Recombinant DVNP binds to double-stranded DNA with similar affinity to histones H2A and H2B in vitro [32]. Furthermore, bioinformatics reveals that DVNP transcripts are amongst the most abundant transcripts in any given dinoflagellate species [95].

Irwin et al. (2018) provided new insights into the possible DVNP-driven evolution of the dinokaryon using *Saccharomyces cerevisiae* as a model. DVNP was expressed in *S. cerevisiae* and the nature of DVNP interaction with its chromatin was assessed. They found that DVNP protein in the yeast nucleus remodels chromatin and preferentially associates with nucleosomal regions leading to nucleosome displacement. As a consequence, transcription is impaired and growth retardation of the affected yeast cells is observed. Remarkably, this effect seems diminished when yeast cells reduce their nuclear histone complement [104]. Irwin et al. hypothesized that the degree of histone depletion seen in dinoflagellates might have been an adaptive response following the acquisition of DVNP through either horizontal gene transfer or following infection with a DVNP-carrying viral pathogen.

Interestingly, a number of studies in the 1970s and 1980s have identified one to two basic proteins with a size range of 10–13 kDa in nuclear extracts of *Gymnodinium nelsonii*, *Gymnodinium dorsum*, *Karenia brevis*, *C. cohnii*, *Peridinium trochoideum*, *P. micans*, and *O. marina* [82,84]. Until recently, all these proteins were thought to represent HLPs, however, with the discovery of DVNPs as a ubiquitous and more ancient feature of dinoflagellates; this needs to be reassessed. Despite growing insights into the potential interactions, origins, and abundance of histones, DVNPs, and HLPs in dinoflagellates, further research is still needed to elucidate the biochemical properties and functional roles of these proteins within the highly aberrant nuclei of dinoflagellates.

## 4. The Dinoflagellate Chromosome Structure Is Unique amongst Eukaryotes

**Figure 4:**
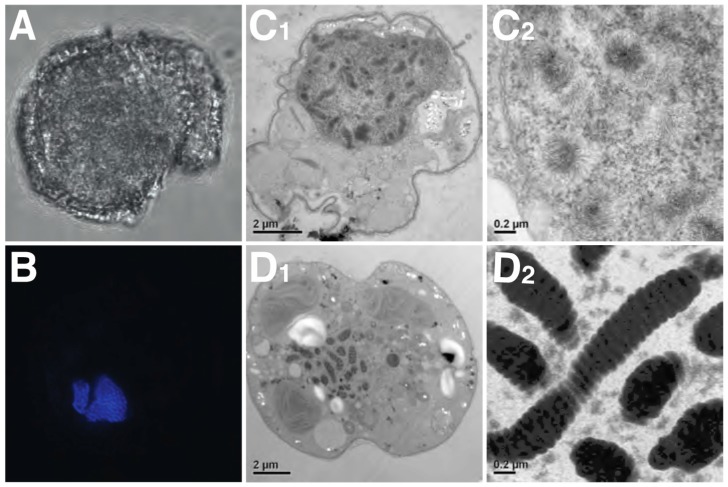
Unusual dinoflagellate chromosome structure. (**A**) Brightfield picture of a formaldehyde–fixed *Karlodinium veneficum* cell. (**B**) Nucleus of above cell stained with DAPI; note the high number and condensed appearance of the chromosomes. (**C_1_**) Electron micrograph of a section of *K. veneficum* cell fixed with glutaraldehyde and stained with osmium tetroxide. Chromosomes have a fluffy, cotton candy-like appearance (**C_2_**). (**D_1_**) A cell from the same culture but high-pressure frozen and freeze substituted with osmium instead. Chromosomes are highly electron dense and compact with a banded pattern (**D_2_**). The differences in appearance of the nuclei and chromosomes in (**C_1_**,**C_2_**) and (**D_1_**,**D_2_**) highlight how diverse fixation protocols produce very disparate results and that interpretation based solely on such pictures is likely not informative.

Chromosome function, telomerase activity, and nucleolar regions (e.g., rDNA sites and nucleolus organizer regions (NORs)) appear to be of a typical eukaryotic nature in dinoflagellates [105,106,107]. Dinoflagellate chromosome structure, on the other hand, is highly unusual (see Figure 4C_1_). Chromosomes are permanently condensed throughout the entire cell cycle, with the exception of a few species such as *N. scintillans* [108] and *Blastodinium* sp. [109,110], which lightly decondense their nuclei during gametogenesis. Although still conspicuous in most taxa, the permanent condensation state of the chromosomes is less evident in some basal dinoflagellate genera (e.g., *N. scintilans, Blastodinium* sp., *Hematodinium* sp., and *O. marina*). This is in stark contrast to the chromosomes of their closest relative *Perkinsus* that are decondensed throughout the non-mitotic stages of the cell cycle in a typical eukaryotic fashion [111]. 

When the DNA within dinoflagellate chromosomes is observed by transmission electron microscopy, an arch-shaped, banded, candy floss-like chromatin morphology is apparent (see Figure 4). This has been linked to the molecular architecture of in vitro cholesteric liquid DNA crystals [112]. It has, therefore, been suggested that in the absence of histone compaction, dinoflagellates rely on these highly dense liquid crystalline states to achieve chromosomal separation necessary during DNA replication and segregation [113]. Indeed, using ATAC-see we can show that, in the basal syndinean dinoflagellate *Hematodinium*, at least two chromatin states exist that appear comparable to accessible euchromatin and inaccessible heterochromatin states found in nuclei of other non-dinoflagellate eukaryotes (see Figure 3A1–A4).

The combination of order and mobility in DNA-based cholesteric liquid crystals might be an essential requirement for self-organization and structure formation in the chromosomes of dinoflagellates [114]. The crystalline formation of the DNA within the dinoflagellate nucleus causes birefringence (or double retardance)—the phase differences between two resulting light rays of a given light path that pass through the liquid crystal [112]. The birefringence of dinoflagellate chromosomes is measurable [115], and it was suggested that it reflects the DNA concentration, the level of condensation and the arrangement of the chromosomes in the nucleus [113], but this remains to be more thoroughly tested. For unknown reasons, some basal dinoflagellate species lack measurable birefringence, however, the orientation of birefringent chromosomes to each other can have additive or subtractive effects on overall birefringence and certain chromosomal alignments could result in cancellation of birefringence [113,115].

Numerous models have been proposed to explain the unusual banded structure of the permanently condensed liquid crystalline chromosomes of dinoflagellates. The four most notable models are: (i) The ‘toroidal chromonema’ model by Okaley and Dodge [116]; (ii) the ‘stacks of DNA discs’ model by Livolant and Bouligand (1980) [77] and Rill et al. (1989) [117]; (iii) the ‘central core fiber’ model by Spector and Trimer (1981) [118]; and (iv) the ‘peeled, cored, sliced pineapple’ model by Levi-Setti et al. (2008) [44]. Levi-Setti et al. used ion probe mass spectroscopy to obtain relatively high-resolution images of whole chromosomes in vivo of three dinoflagellate species and observed that the axial regions of the chromosomes were devoid of Mg^2+^ and Ca^2+^ ions, suggesting a protein-rich rather than a DNA-rich core [44]. However, considerable uncertainty of the nature and manner of organization of these highly structured chromosomes remains.

In eukaryotes other than dinoflagellates, chromosomes are ‘classically’ described as DNA (2 nm) wrapped around a core of histones forming a 11 nm ‘beads on a string’ nucleosome fiber, which in turn forms a highly organized 30 nm chromatin fiber that then is packed into its higher-order chromosomal form. However, the existence of the 30 nm fiber is still controversial [43,119,120,121,122,123,124]. As more and more data have accumulated, it has emerged that the 30 nm fibers represent a common fixation artifact caused by chromosomal swelling under low-salt conditions and consecutive loss of inter-fiber associations of the 11 nm nucleosomal ‘beads on a string’ fiber [125]. Generally, 30 nm fibers occur only under special circumstances [122,126], when intra-fiber nucleosomal associations dominate over inter-fiber associations within chromosomes [127]. In a densely packed in vivo nucleus, the 11 nm fibers are irregularly folded onto a condensin scaffold instead [125,128]. The nucleosome–DNA complexes are further compacted through inter-fiber nucleosome associations like random polymer chains, which move and rearrange constantly in a highly disordered state [125,127]. The fact that the 30 nm fiber model is now slowly replaced by more complex models of eukaryotic chromatin organization highlights that even in the better-studied eukaryotic model systems much still has to be learned about nuclear and chromosomal biochemistry [129,130].

Compared with the more static 30 nm model, the new, more dynamic models of chromatin compaction enable a new perspective on hypotheses of the transition from a nucleosome-based chromatin structure of a dinoflagellate ancestor to chromatin that seems to be mostly histone-free in extant dinoflagellate species. It has been shown recently that heterochromatin protein 1 alpha (HP1α), which is commonly associated with silenced heterochromatic regions in many eukaryotes, can form phase-separated droplets when phosphorylated or bound to DNA [131]. Nucleosomes and DNA itself preferentially partition into such HP1α droplets both in vitro and in vivo. Based on these, the authors speculate that heterochromatin-mediated gene silencing may occur in part through this sequestration of compacted chromatin [131]. DVNPs and HLPs may play a similar role in dinoflagellate chromatin, and it is easy to envisage that an expansion of the presence and properties of such phase-separated compartments might have been instrumental in the development of the dinokaryotic state. Future research that investigates the role and nature of phase separation and liquid crystals in dinoflagellate chromatin is clearly warranted. 

It is of note that the sequence and nucleotide-modified state of dinoflagellate genomes are not inherently incompatible with canonical nucleosomal compaction. Liu et al. injected chromosomes isolated from *C. cohnii* into a cell-free system derived from *Xenopus laevis* eggs [132]. The dinoflagellate chromosomes first decondensed and then recondensed in a typical eukaryotic fashion without liquid crystalline properties. A nuclear envelope was also assembled, and the nucleus was apparently fully reconstituted as a typical eukaryotic nucleus. This indicates that dinoflagellate genomic structure, including nucleotide modifications, does not preclude typical chromosomal arrangements. Rather, it is likely the protein components of the nucleus that are chiefly responsible for coordinating the dinokaryotic state.

## 5. Stepwise Evolution of the Aberrant Dinoflagellate Nucleus

Drawing on the advances in knowledge of both the phylogenetic history of dinoflagellates and the biochemical properties of their nuclei, we now are able to hypothesize the stepwise evolutionary history of the deviant dinoflagellate nuclei with much more certainty (see Figure 1).

The closest relative to the dinoflagellates, *Perkinsus* still possesses a canonical nucleus—it compacts its DNA using nucleosomes with typical histones similar to related cells such as *P. falciparum*. It lacks the novel nucleoproteins DVNPs and HLPs. However, in the early branching dinoflagellate, *O. marina*, histones are in low abundance and DVNPs become dominant features of the dinoflagellate nucleus [32,85]. Furthermore, while *Perkinsus* species possess small genomes (28–46 Mb) [32], *O. marinas* has inflated its genome to a massive 55 Gb [133]. *Hematodinium* sp. similarly lacks bulk nucleosomal DNA condensation with DVNPs the dominant basic nucleoproteins (see Figure 3B1–B4) and has an extensive genome at approximately 4.8 Gb [32].

The dramatic change seen between *Perkinsus* and these early -branching dinoflagellates suggests that gain of DVNP was a pivotal event in nuclear remodeling. The predicted antiquity of the viruses in which DVNP is found [134,135,136] implies that the lateral gain of DVNP likely occurred from virus to dinoflagellate, although promiscuous transfer of genes between viruses confound any clear interpretation of these events. The function of DVNPs in the viruses is also unknown, but it is conspicuous that the viruses that possess this protein also have the largest of viral genomes, so a role in viral genome compaction seems credible. A dual role as a virulence factor is also possible, and indeed DVNP proteins expressed in other eukaryotic systems show toxicity through interruption of chromatin function [104]. One possibility is that dinoflagellates gained immunity against phycodnaviral infection by adapting to accommodate DVNP in their nuclei. In any case, the timing of DVNP gain seems to correlate with massive expansion in dinoflagellate genome size [2,30,31], although the basis of this correlation is unclear. Perhaps the high density of DVNP-driven DNA compaction allows genome expansion to maintain functional regions at the chromosome surfaces, or perhaps the loss of the nucleosomal histones as bulk packaging agents disrupted genome size control. DNA replication in eukaryotes is tightly coupled with nucleosome assembly and both mechanisms regulate each other [65]. The loss of nucleosomes might have decoupled these processes promoting iterative genome duplication events. In other eukaryotes, non-histone DNA condensation mechanisms exist, such as in sperm nuclei of a variety of metazoan species where specific histone variants, protamines, and other lysine-rich small proteins displace bulk nucleosomes [62,137,138]. However, these states are temporary and render the nucleus transcriptionally inactive, so are likely not functionally analogous to the DVNP-form of chromatin management.

A second group of dinoflagellate specific nuclear proteins, the bacterial HU-like proteins HLP-I and HLP-II, are only found in core dinoflagellates after the divergence of the Syndiniaceae [2,97]. These proteins were acquired in two separate events, HLP-II first, which was then displaced in many lineages by HLP-I [2]. These HLPs appear to have provided a new layer of chromatin remodeling as it is in these taxa where a more conspicuous liquid crystalline presentation of dinokaryon chromosomes is evident. Thus, we can establish that at least three independent waves of protein gain lead to the unique architecture of the nucleus of core dinoflagellates. 

Displacement of histones as the bulk DNA packaging agents has evidently not displaced all histone functions. It remains unknown what functions dinoflagellate histones now perform but, given their relatively low abundance, it seems likely that these might include similar functions to the histone variants such as kinetochore assembly, DNA-damage responses, and perhaps even transcriptional control and epigenetics. The divergence of histone sequences, even more so than within variants of other eukaryotes, is difficult to account for, but perhaps this reflects a freedom of constraints imposed by variants having to interact with the classic core histones. In any case, it is apparent that histones retain a place in dinokaryon function despite their many differences from conventional nuclei.

## 6. Concluding Remarks

Work is now required to elucidate further the role of DVNPs, HLPs, and histones in the dinoflagellate chromatin and nuclear machinery. Established techniques that enable the elucidation of the genomic conformation, availability, and protein occupancy, such as Hi-C-type chromosome conformation capture techniques [139], Assay for Transposase-Accessible Chromatin (ATAC)-seq/see (Figure 4A; [140]), and chromatin immunoprecipitation (ChIP)-seq [141], are now within reach of dinoflagellate research and should help provide answers to many outstanding questions. The function of nucleotide modifications within the dinokaryotic state also remains unknown, including its sequence of appearance within the progression of dinokaryon evolution. It is currently unknown whether such modifications exist or play important roles in basal taxa such as *Hematodinium* sp. or *Perkinsus*. The presence, significance and role of divalent cations and transition metals also awaits elucidation. Moreover, Okamoto et al. (2012) described two new basal dinoflagellate species, *Psammosa pacifica* and *Psammosa atlantica*, which diverged between the genus *Perkinsus* and *O. marina* [142]. It may be that these will provide further insight into the early events that resulted in the dinokaryon once the characters of these taxa’s nuclei are known. It is clear that dinoflagellates challenge our models of eukaryotic nuclei. This is very timely as the dynamic, heterogenous nature of all eukaryotic nuclei is also currently undergoing something of a revision [129].

## References

[1] De Vargas C., Audic S., Henry N., Decelle J., Mahé F., Logares R., Lara E., Berney C., Le Bescot N., Probert I. (2015). Ocean plankton. Eukaryotic plankton diversity in the sunlit ocean. Science.

[2] Janouškovec J., Gavelis G.S., Burki F., Dinh D., Bachvaroff T.R., Gornik S.G., Bright K.J., Imanian B., Strom S.L., Delwiche C.F. (2017). Major transitions in dinoflagellate evolution unveiled by phylotranscriptomics. Proc. Natl. Acad. Sci. USA.

[3] Jackson C.J., Gornik S.G., Waller R.F. (2012). The mitochondrial genome and transcriptome of the basal dinoflagellate *Hematodinium* sp.: Character evolution within the highly derived mitochondrial genomes of dinoflagellates. Genome Biol. Evol..

[4] Danne J.C., Gornik S.G., MacRae J.I., McConville M.J., Waller R.F. (2013). Alveolate mitochondrial metabolic evolution: Dinoflagellates force reassessment of the role of parasitism as a driver of change in apicomplexans. Mol. Biol. Evol..

[5] Hackett J.D., Anderson D.M., Erdner D.L., Bhattacharya D. (2004). Dinoflagellates: A remarkable evolutionary experiment. Am. J. Bot..

[6] Gómez F. (2012). A quantitative review of the lifestyle, habitat and trophic diversity of dinoflagellates (Dinoflagellata, Alveolata). Syst. Biodivers..

[7] Dodge J.D. (1983). Dinoflagellates: Investigation and phylogenetic speculation. Br. Phycol. J..

[8] Bachvaroff T.R., Handy S.M., Place A.R., Delwiche C.F. (2011). Alveolate phylogeny inferred using concatenated ribosomal proteins. J. Euk. Microbiol..

[9] Bachvaroff T.R., Gornik S.G., Concepcion G.T., Waller R.F., Mendez G.S., Lippmeier J.C., Delwiche C.F. (2014). Dinoflagellate phylogeny revisited: Using ribosomal proteins to resolve deep branching dinoflagellate clades. Mol. Phylogenet. Evol..

[10] Fensome R.A., Saldarriaga J.F., Taylor M.F.J.R. (1999). Dinoflagellate phylogeny revisited: Reconciling morphological and molecular based phylogenies. Grana.

[11] Saldarriaga J.F., Taylor F.J., Keeling P.J., Cavalier-Smith T. (2001). Dinoflagellate nuclear SSU rRNA phylogeny suggests multiple plastid losses and replacements. J. Mol. Evol..

[12] Zhang H., Bhattacharya D., Lin S. (2005). Phylogeny of dinoflagellates based on mitochondrial cytochrome B and nuclear small subunit rDNA sequence comparisons. J. Phycol..

[13] Shalchian-Tabrizi K., Minge M.A., Cavalier-Smith T., Nedreklepp J.M., Klaveness D., Jakobsen K.S. (2006). Combined heat shock protein 90 and ribosomal RNA sequence phylogeny supports multiple replacements of dinoflagellate plastids. J. Euk. Microbiol..

[14] Hoppenrath M., Leander B.S. (2010). Dinoflagellate phylogeny as inferred from heat shock protein 90 and ribosomal gene sequences. PLoS ONE.

[15] Cavalier-Smith T., Chao E.E. (2004). Protalveolate phylogeny and systematics and the origins of Sporozoa and dinoflagellates (phylum Myzozoa nom. nov.). Europ. J. Protistol..

[16] Adl S.M., Bass D., Lane C.E., Lukeš J., Schoch C.L., Smirnov A., Agatha S., Berney C., Brown M.W., Burki F. (2019). Revisions to the classification, nomenclature, and diversity of eukaryotes. J. Euk. Microbiol..

[17] Adl S.M., Simpson A.G.B., Farmer M.A., Andersen R.A., Anderson O.R., Barta J.R., Bowser S.S., Brugerolle G., Fensome R.A., Fredericq S. (2005). The new higher level classification of eukaryotes with emphasis on the taxonomy of protists. J. Euk. Microbiol..

[18] Adl S.M., Simpson A.G.B., Lane C.E., Lukeš J., Bass D., BowserR S.S., Brown M.W., Burki F., Dunthorn M., Hampl V. (2012). The revised classification of eukaryotes. J. Euk. Microbiol..

[19] DeBarry J.D., Kissinger J.C. (2011). Jumbled genomes: Missing apicomplexan synteny. Mol. Biol. Evol..

[20] Cavalier-Smith T. (2005). Economy, speed and size matter: Evolutionary forces driving nuclear genome miniaturization and expansion. Ann. Bot..

[21] Striepen B., Jordan C.N., Reiff S., van Dooren G.G. (2007). Building the perfect parasite: Cell division in Apicomplexa. PLoS Pathog..

[22] Wisecaver J.H., Hackett J.D. (2011). Dinoflagellate Genome Evolution. Annu. Rev. Microbiol..

[23] Jaeckel-Williams R. (1978). Nuclear divisions with reduced numbers of microtubules in *Tetrahymena*. J. Cell. Sci..

[24] Jenkins R.A. (1967). Fine structure of division in ciliate protozoa. I. Micronuclear mitosis in *Blepharisma*. J. Cell Biol..

[25] Carvalho-Santos Z., Azimzadeh J., Pereira-Leal J.B., Bettencourt-Dias M. (2011). Evolution: Tracing the origins of centrioles, cilia, and flagella. J. Cell Biol..

[26] Ausseil J., Soyer-Gobillard M.-O., Géraud M.-L., Bhaud Y., Perret E., Barbier M., Albert M., Plaisance L., Moreau H. (2000). Dinoflagellate centrosome: Associated proteins old and new. Europ. J. Protistol..

[27] Triemer R.E. (1982). A Unique mitotic variation in the marine dinoflagellate *Oxyrrhis marina* (Pyrrophyta). J. Phycol..

[28] Ris H., Kubai D.F. (1974). An unusual mitotic mechanism in the parasitic protozoan *Syndinium* sp.. J. Cell Biol..

[29] Keeling P.J., Slamovits C.H. (2005). Causes and effects of nuclear genome reduction. Curr. Opin. Genet. Dev..

[30] LaJeunesse T.C., Lambert G., Andersen R.A., Coffroth M.A., Galbraith D.W. (2005). *Symbiodinium* (Pyrrhophyta) genome sizes (DNA content) are smallest among dinoflagellates. J. Phycol..

[31] Lin S. (2006). The smallest dinoflagellate genome is yet to be found: A comment on LaJeunesse et al. “*Symbiodinium* (Pyrrhophyta) genome sizes (DNA content) are smallest among dinoflagellates”. J. Phycol..

[32] Gornik S.G., Ford K.L., Mulhern T.D., Bacic A., McFadden G.I., Waller R.F. (2012). Loss of nucleosomal DNA condensation coincides with appearance of a novel nuclear protein in dinoflagellates. Curr. Biol..

[33] Khandelwal S. (1990). Chromosome Evolution in the Genus *Ophioglossum*. Bot. J. Lin. Soc..

[34] Zhou L., Gui J.F. (2002). Karyotypic diversity in polyploid gibel carp, *Carassius auratus gibelio* Bloch. Genetica.

[35] Contreras L.C., Torres-Mura J.C., Spotorno A.E. (1990). The largest known chromosome number for a mammal, in a South American desert rodent. Experientia.

[36] Gardner M.J., Hall N., Fung E., White O., Berriman M., Hyman R.W., Carlton J.M., Pain A., Nelson K.E., Bowman S. (2002). Genome sequence of the human malaria parasite *Plasmodium falciparum*. Nature.

[37] Jones S.H., Lew A.E., Jorgensen W.K., Barker S.C. (1997). *Babesia bovis*: Genome size, number of chromosomes and telomeric probe hybridisation. Int. J. Parasitol..

[38] Brayton K.A., Lau A.O.T., Herndon D.R., Hannick L., Kappmeyer L.S., Berens S.J., Bidwell S.L., Brown W.C., Crabtree J., Fadrosh D. (2007). Genome Sequence of *Babesia bovis* and Comparative Analysis of Apicomplexan Hemoprotozoa. PLoS Pathog..

[39] Leonor Teles-Grilo M., Duarte S.M., Tato-Costa J., Gaspar-Maia A., Oliveira C., Rocha A.A., Marques A., Cordeiro-da-Silva A., Azevedo C. (2007). Molecular karyotype analysis of *Perkinsus atlanticus* (Phylum Perkinsozoa) by pulsed field gel electrophoresis. Europ. J. Protistol..

[40] Marques A.R., Tato-Costa J., Conde C., Azevedo C., Teles-Grilo M.L. (2012). Chromosomal localisation of five genes in *Perkinsus olseni* (Phylum Perkinsozoa). Europ. J. Protistol..

[41] Herzog M., Soyer M.O. (1983). The native structure of dinoflagellate chromosomes and their stabilization by Ca^2+^ and Mg^2+^ cations. Europ. J. Cell Biol..

[42] Strick R., Strissel P.L., Gavrilov K., Levi-Setti R. (2001). Cation–chromatin binding as shown by ion microscopy is essential for the structural integrity of chromosomes. J. Cell Biol..

[43] Woodcock C.L., Ghosh R.P. (2010). Chromatin higher-order structure and dynamics. Cold Spring Harb. Perspect. Biol..

[44] Levi-Setti R., Gavrilov K., Rizzo P. (2008). Divalent cation distribution in dinoflagellate chromosomes imaged by high-resolution ion probe mass spectrometry. Europ. J Cell Biol..

[45] Koltover I., Wagner K., Safinya C.R. (2000). DNA condensation in two dimensions. Proc. Nat. Acad. Sci. USA.

[46] Kearns L.P., Sigee D.C. (1980). The occurrence of period IV elements in dinoflagellate chromatin: An X-ray microanalytical study. J. Cell. Sci..

[47] Korlach J., Turner S.W. (2012). Going beyond five bases in DNA sequencing. Curr. Opin. Struct. Biol..

[48] Breiling A., Lyko F. (2015). Epigenetic regulatory functions of DNA modifications: 5-methylcytosine and beyond. Epigenetics Chromatin.

[49] Kumar S., Chinnusamy V., Mohapatra T. (2018). Epigenetics of modified DNA bases: 5-methylcytosine and beyond. Front. Genet..

[50] Gommers-Ampt J.H., Borst P. (1995). Hypermodified bases in DNA. FASEB J..

[51] Cadet J., Bellon S., Douki T., Frelon S., Gasparutto D., Muller E., Pouget J.-P., Ravanat J.-L., Romieu A., Sauvaigo S. (2004). Radiation-induced DNA damage: Formation, measurement, and biochemical features. J. Environ. Pathol. Toxicol. Oncol..

[52] Kallen R.G., Simon M., Marmur J. (1962). The occurrence of a new pyrimidine base replacing thymine in a bacteriophage DNA: 5-hydroxymethyl uracil. J. Mol. Biol..

[53] Wu X., Zhang Y. (2017). TET-mediated active DNA demethylation: Mechanism, function and beyond. Nat. Rev. Genet..

[54] Greene J.R., Morrissey L.M., Foster L.M., Geiduschek E.P. (1986). DNA binding by the bacteriophage SPO1-encoded type II DNA-binding protein, transcription factor 1. Formation of nested complexes at a selective binding site. J. Biol. Chem..

[55] Rae P.M.M. (1973). 5-Hydroxymethyluracil in the DNA of a dinoflagellate. Proc. Nat. Acad. Sci. USA.

[56] Rae P. (1976). Hydroxymethyluracil in eukaryote DNA: A natural feature of the pyrrophyta (dinoflagellates). Science.

[57] Davies W., Jakobsen K.S., Nordby O. (1988). Characterization of DNA from the dinoflagellate *Woloszynskia bostoniensis*. J. Protozool..

[58] Blank R.J., Huss V.A.R., Kersten W. (1988). Base composition of DNA from symbiotic dinoflagellates: A tool for phylogenetic classification. Arch. Microbiol..

[59] Steele R.E., Rae P.M.M. (1980). Ordered distribution of modified bases in the DNA of a dinoflagellate. Nucleic Acids Res..

[60] Rae P.M.M., Steele R.E. (1978). Modified bases in the DNAs of unicellular eukaryotes: An examination of distributions and possible roles, with emphasis on hydroxymethyluracil in dinoflagellates. Biosystems.

[61] De Mendoza A., Bonnet A., Vargas-Landin D.B., Ji N., Hong F., Yang F., Li L., Hori K., Pflueger J., Buckberry S. (2018). Recurrent acquisition of cytosine methyltransferases into eukaryotic retrotransposons. Nat. Commun..

[62] Talbert P.B., Henikoff S. (2010). Histone variants—Ancient wrap artists of the epigenome. Nat. Rev. Mol. Cell. Biol..

[63] Davie J.R., Lin R., Allis C.D. (1991). Timing of the appearance of ubiquitinated histones in developing new macronuclei of *Tetrahymena thermophila*. Biochem. Cell Biol..

[64] Miao J., Fan Q., Cui L., Li J., Li J., Cui L. (2006). The malaria parasite *Plasmodium falciparum* histones: Organization, expression, and acetylation. Gene.

[65] Burgess R.J., Zhang Z. (2013). Histone chaperones in nucleosome assembly and human disease. Nat. Struct. Mol. Biol..

[66] Jenuwein T., Allis C.D. (2001). Translating the histone code. Science.

[67] Strahl B.D., Allis C.D. (2000). The language of covalent histone modifications. Nature.

[68] Talbert P.B., Ahmad K., Almouzni G., Ausió J., Berger F., Bhalla P.L., Bonner W.M., Cande W., Chadwick B.P., Chan S.W.L. (2012). A unified phylogeny-based nomenclature for histone variants. Epigenetics Chromatin.

[69] Santaguida S., Musacchio A. (2009). The life and miracles of kinetochores. EMBO J..

[70] Zlatanova J., Thakar A. (2008). H2A.Z: View from the top. Structure.

[71] Petter M., Lee C.C., Byrne T.J., Boysen K.E., Volz J., Ralph S.A., Cowman A.F., Brown G.V., Duffy M.F. (2011). Expression of *P. falciparum var* genes involves exchange of the histone variant H2A.Z at the promoter. PLoS Pathog..

[72] van Attikum H., Gasser S.M. (2009). Crosstalk between histone modifications during the DNA damage response. Trends Cell Biol..

[73] Ng R.K., Gurdon J.B. (2007). Epigenetic memory of an active gene state depends on histone H3.3 incorporation into chromatin in the absence of transcription. Nat. Cell Biol..

[74] Marzluff W.F., Koreski K.P. (2017). Birth and Death of Histone mRNAs. Trends Genet..

[75] Marzluff W.F., Wagner E.J., Duronio R.J. (2008). Metabolism and regulation of canonical histone mRNAs: Life without a poly(A) tail. Nat. Rev. Genet..

[76] Livolant F., Bouligand Y. (1978). New observations on the twisted arrangement of dinoflagellate chromosomes. Chromosoma.

[77] Livolant F., Bouligand Y. (1980). Double helical arrangement of spread dinoflagellate chromosomes. Chromosoma.

[78] Rizzo P.J., Burghardt R.C. (1980). Chromatin structure in the unicellular algae *Olisthodiscus luteus*, *Crypthecodinium cohnii* and *Peridinium balticum*. Chromosoma.

[79] Bodansky S., Mintz L.B., Holmes D.S. (1979). The mesokaryote *Gyrodinium cohnii* lacks nucleosomes. Biochem. Biophys. Res. Commun..

[80] Rizzo P.J. (2003). Those amazing dinoflagellate chromosomes. Cell Res..

[81] Rizzo P.J. (1987). Biochemistry of the dinoflagellate nucleus. The Biology of Dinoflagellates.

[82] Rizzo P.J., Noodén L.D. (1972). Chromosomal Proteins in the Dinoflagellate Alga *Gyrodinium cohnii*. Science.

[83] Rizzo P.J., Burghardt R.C. (1982). Histone-like protein and chromatin structure in the wall-less dinoflagellate *Gymnodinium nelsoni*. Biosystems.

[84] Herzog M., Soyer M.O. (1981). Distinctive features of dinoflagellate chromatin. Absence of nucleosomes in a primitive species *Prorocentrum micans* E. Europ. J. Cell Biol..

[85] Kato K.H., Moriyama A., Huitorel P., Cosson J., Cachon M., Sato H. (1997). Isolation of the major basic nuclear protein and its localization on chromosomes of the dinoflagellate, *Oxyrrhis marina*. Biol. Cell.

[86] Vernet G., Salarovira M., Maeder M., Jacques F., Herzog M. (1990). Basic Nuclear Proteins of the Histone-Less Eukaryote *Crypthecodinium cohnii* (Pyrrhophyta)—2-dimensional electrophoresis and DNA-binding properties. Biochim. Biophys. Acta.

[87] Dodge J.D. (1964). Chromosome structure in the dinophyceae. Arch. Microbiol..

[88] Okamoto O.K., Hastings J.W. (2003). Genome-wide analysis of redox-regulated genes in a dinoflagellate. Gene.

[89] Hackett J.D., Scheetz T.E., Yoon H.S., Soares M.B., Bonaldo M.F., Casavant T.L., Bhattacharya D. (2005). Insights into a dinoflagellate genome through expressed sequence tag analysis. BMC Genomics.

[90] Roy S., Morse D. (2012). A Full suite of histone and histone modifying genes are transcribed in the dinoflagellate *Lingulodinium*. PLoS ONE.

[91] Bayer T., Aranda M., Sunagawa S., Yum L.K., DeSalvo M.K., Lindquist E., Coffroth M.A., Voolstra C.R., Medina M. (2012). *Symbiodinium* transcriptomes: Genome insights into the dinoflagellate symbionts of reef-building corals. PLoS ONE.

[92] Riaz S., Sui Z. (2018). Molecular Cloning, Transcriptome profiling, and characterization of histone genes in the dinoflagellate *Alexandrium pacificum*. J. Microbiol. Biotechnol..

[93] Lin S., Zhang H., Zhuang Y., Tran B., Gill J., Hastings W. (2010). Spliced leader-based metatranscriptomic analyses lead to recognition of hidden genomic features in dinoflagellates. Proc. Natl. Acad. Sci. USA.

[94] Zhang H., Hou Y., Miranda L., Campbell D.A., Sturm N.R., Gaasterland T., Lin S. (2007). Spliced leader RNA trans-splicing in dinoflagellates. Proc. Natl. Acad. Sci. USA.

[95] Marinov G.K., Lynch M. (2016). Diversity and divergence of dinoflagellate histone proteins. G3.

[96] Cui B., Liu Y., Gorovsky M.A. (2006). Deposition and function of histone H3 variants in *Tetrahymena thermophila*. Mol. Cell. Biol..

[97] Wong J.T.Y., New D.C., Wong J.C.W., Hung V.K.L. (2003). Histone-like proteins of the dinoflagellate *Crypthecodinium cohnii* have homologies to bacterial DNA-binding proteins. Eukaryot. Cell.

[98] Sala-Rovira M., Geraud M.L., Caput D., Jacques F., Soyer-Gobillard M.O., Vernet G., Herzog M. (1991). Molecular cloning and immunolocalization of two variants of the major basic nuclear protein (HCc) from the histone-less eukaryote *Crypthecodinium cohnii* (Pyrrhophyta). Chromosoma.

[99] Geraud M.L., Salarovira M., Herzog M., Soyer-Gobillard M.O. (1991). Immunocytochemical localization of the DNA-binding protein HCc during the cell-cycle of the histone-less dinoflagellate Protoctista *Crypthecodinium cohnii* B. Biol. Cell.

[100] Chan Y.-H., Wong J.T.Y. (2007). Concentration-dependent organization of DNA by the dinoflagellate histone-like protein HCc3. Nucleic Acids Res..

[101] Sun S., Liu M., Dong F., Fan S., Yao Y. (2013). A Histone-Like protein induces plasmid DNA to form liquid crystals in vitro and gene compaction in vivo. Int. J. Mol. Sci..

[102] Chudnovsky Y., Li J.F., Rizzo P.J., Hastings J.W., Fagan T.F. (2002). Cloning, expression, and characterization of a histone-like protein from the marine dinoflagellate *Lingulodinium polyedrum* (Dinophyceae). J. Phycol..

[103] Taroncher-Oldenburg G., Anderson D.M. (2000). Identification and characterization of three differentially expressed genes, encoding S-adenosylhomocysteine hydrolase, methionine aminopeptidase, and a histone-like protein, in the toxic dinoflagellate *Alexandrium fundyense*. Appl. Environ. Microbiol..

[104] Irwin N.A.T., Martin B.J.E., Young B.P., Browne M.J.G., Flaus A., Loewen C.J.R., Keeling P.J., Howe L.J. (2018). Viral proteins as a potential driver of histone depletion in dinoflagellates. Nat. Commun..

[105] Alverca E., Franca S., Díaz de la Espina S.M. (2006). Topology of splicing and snRNP biogenesis in dinoflagellate nuclei. Biol. Cell.

[106] Alverca E., Cuadrado A., Jouve N., Franca S., Moreno Díaz de la Espina S. (2007). Telomeric DNA localization on dinoflagellate chromosomes: Structural and evolutionary implications. Cytogenet. Genome Res..

[107] Fojtová M., Wong J.T.Y., Dvořáčková M., Yan K.T.H., Sýkorová E., Fajkus J. (2010). Telomere maintenance in liquid crystalline chromosomes of dinoflagellates. Chromosoma.

[108] Fukuda Y., Endoh H. (2006). New details from the complete life cycle of the red-tide dinoflagellate *Noctiluca scintillans* (Ehrenberg) McCartney. Europ. J. Protistol..

[109] Skovgaard A., Karpov S.A., Guillou L. (2012). The parasitic dinoflagellates *Blastodinium* spp. inhabiting the gut of marine, planktonic copepods: Morphology, ecology, and unrecognized species diversity. Front. Microbiol..

[110] Soyer M.O. (1971). Structure du noyau des *Blastodinium* (Dinoflagellés parasites). Chromosoma.

[111] Perkins F.O., Menzel R.W. (1967). Ultrastructure of sporulation in the oyster pathogen *Dermocystidium marinum*. J. Invert. Path..

[112] Bouligand Y. (1972). Twisted fibrous arrangements in biological materials and cholesteric mesophases. Tissue Cell.

[113] Chow M.H., Yan K.T.H., Bennett M.J., Wong J.T.Y. (2010). Birefringence and DNA condensation of liquid crystalline chromosomes. Eukaryot. Cell.

[114] Mitov M. (2017). Cholesteric liquid crystals in living matter. Soft Matter.

[115] Cachon J., Sato H., Cachon M., Sato Y. (1989). Analysis by polarizing microscopy of chromosomal structure among dinoflagellates and its phylogenetic involvement. Biol. Cell.

[116] Oakley B.R., Dodge J.D. (1979). Evidence for a double-helically coiled toroidal chromonema in the dinoflagellate chromosome. Chromosoma.

[117] Rill R.L., Livolant F., Aldrich H.C., Davidson M.W. (1989). Electron-microscopy of liquid-crystalline DNA—Direct evidence for cholesteric-like organization of DNA in dinoflagellate chromosomes. Chromosoma.

[118] Spector D.L., Triemer R.E. (1981). Chromosome structure and mitosis in the dinoflagellates—An ultrastructural approach to an evolutionary problem. Biosystems.

[119] Grigoryev S.A., Woodcock C.L. (2012). Chromatin organization—The 30nm fiber. Exp. Cell Res..

[120] Horowitz-Scherer R.A., Woodcock C.L. (2005). Organization of interphase chromatin. Chromosoma.

[121] Eltsov M., MacLellan K.M., Maeshima K., Frangakis A.S., Dubochet J. (2008). Analysis of cryo-electron microscopy images does not support the existence of 30 nm chromatin fibers in mitotic chromosomes in situ. Proc. Natl. Acad. Sci. USA.

[122] Scheffer M.P., Eltsov M., Frangakis A.S. (2011). Evidence for short-range helical order in the 30 nm chromatin fibers of erythrocyte nuclei. Proc. Natl. Acad. Sci. USA.

[123] Razin S.V., Gavrilov A.A. (2014). Chromatin without the 30 nm fiber: Constrained disorder instead of hierarchical folding. Epigenetics.

[124] Cai S., Song Y., Chen C., Shi J., Gan L. (2018). Natural chromatin is heterogeneous and self-associates in vitro. Mol. Biol. Cell.

[125] Maeshima K., Hihara S., Takata H. (2011). New insight into the mitotic chromosome structure: Irregular folding of nucleosome fibers without 30 nm chromatin structure. Cold Spring Harb. Symp. Quant. Biol..

[126] Woodcock C.L. (1994). Chromatin fibers observed in situ in frozen hydrated sections. Native fiber diameter is not correlated with nucleosome repeat length. J. Cell. Biol..

[127] Nishino Y., Eltsov M., Joti Y., Ito K., Takata H., Takahashi Y., Hihara S., Frangakis A.S., Imamoto N., Ishikawa T. (2012). Human mitotic chromosomes consist predominantly of irregularly folded nucleosome fibres without a 30 nm chromatin structure. EMBO J..

[128] Joti Y., Hikima T., Nishino Y., Kamda F., Hihara S., Takata H., Ishikawa T., Maeshima K. (2012). Chromosomes without a 30 nm chromatin fiber. Nucleus..

[129] Watson M., Stott K. (2019). Disordered domains in chromatin-binding proteins. Essays Biochem..

[130] Wu C., Travers A. (2019). Modelling and DNA topology of compact 2-start and 1-start chromatin fibres. Nucleic Acids Res..

[131] Larson A.G., Elnatan D., Keenen M.M., Trnka M.J., Johnston J.B., Burlingame A.L., Agard D.A., Redding S., Narlikar G.J. (2017). Liquid droplet formation by HP1α suggests a role for phase separation in heterochromatin. Nature.

[132] Liu X.L., Yan S.H.E.N., Chen E.J., Zhai Z.H. (2000). Nuclear assembly of purified *Crythecodinium cohnii* chromosomes in cell-free extracts of *Xenopus laevis* eggs. Cell Res..

[133] Sano J., Kato K.H. (2009). Localization and copy number of the protein-coding genes actin, α-tubulin and Hsp90 in the nucleus of a primitive dinoflagellate, *Oxyrrhis marina*. Zool. Sci..

[134] Van Etten J.L., Graves M.V., Müller D.G., Boland W., Delaroque N. (2002). Phycodnaviridae—large DNA algal viruses. Arch. Virol..

[135] Yutin N., Colson P., Raoult D., Koonin E.V. (2013). Mimiviridae: Clusters of orthologous genes, reconstruction of gene repertoire evolution and proposed expansion of the giant virus family. Virol. J..

[136] Dunigan D.D., Fitzgerald L.A., Van Etten J.L. (2006). Phycodnaviruses: A peek at genetic diversity. Virus Res..

[137] Török A., Schiffer P.H., Schnitzler C.E., Ford K., Mullikin J.C., Baxevanis A.D., Bacic A., Frank U., Gornik S.G. (2016). The cnidarian *Hydractinia echinata* employs canonical and highly adapted histones to pack its DNA. Epigenetics Chromatin.

[138] Török A., Gornik S.G. (2018). Sperm nuclear basic proteins of marine invertebrates. Results Probl. Cell Differ..

[139] Eagen K.P. (2018). Principles of chromosome architecture revealed by Hi-C. Trends Biochem. Sci..

[140] Chen X., Shen Y., Draper W., Buenrostro J.D., Litzenburger U., Cho S.W., Satpathy A.T., Carter A.C., Ghosh R.P., East-Seletsky A. (2016). ATAC-see reveals the accessible genome by transposase-mediated imaging and sequencing. Nat. Methods.

[141] Furey T.S. (2012). ChIP-seq and beyond: New and improved methodologies to detect and characterize protein-DNA interactions. Nat. Rev. Genet..

[142] Okamoto N., Horák A., Keeling P.J. (2012). Description of two species of early branching dinoflagellates, *Psammosa pacifica* n. g., n. sp. and *P. atlantica* n. sp.. PLoS ONE.

